# Expression of Concern: MiR-30e-5p inhibits the migration and invasion of nasopharyngeal carcinoma via regulating the expression of MTA1

**DOI:** 10.1042/BSR-20194309_EOC

**Published:** 2021-04-07

**Keywords:** Invasion, Migration, MiR-30e-5p, MTA1, Nasopharyngeal carcinoma

The Editorial Office has been made aware of potential issues surrounding the scientific validity of this paper, hence has issued an expression of concern to notify readers whilst the Editorial Office investigates.

